# Peridontopathogenic key species in correlation to the current classification system

**DOI:** 10.1007/s00784-025-06413-2

**Published:** 2025-06-12

**Authors:** Bernd Sigusch, Stefan Kranz, Markus Heyder, Anna Weber, André Güllmar, Nargis Mahmudova, Markus Reise

**Affiliations:** Department of Conservative Dentistry and Periodontology, Center of Dental Medicine, Jena University Hospitals, An der alten Post 4, 07743 Jena, Germany

**Keywords:** Aggregatibacter actinomycetemcomitans, Dysbiosis, Oral bacteria, Periodontitis, Periodontitis classification, Porphyromonas gingivalis, Advanced periodontitis

## Abstract

**Objective:**

The present in-vivo-study aimed on identifying periodontopathogenic key species in correlation to the current classification system. It was evaluated if there is an association in frequency to single periodontitis stages and grades.

**Materials and methods:**

The study included 100 patients that were assigned to 4 test groups (periodontitis stages I-IV) and one healthy control group, each *n* = 20. Samples were collected from the deepest pockets of each sextant and analyzed for: *Fusobacterium nucleatum* (*F.n.*), *Aggregatibacter actinomycetemcomitans* (*A.a.*), *Porphyromonas gingivalis* (*P.g.*), *Tannerella forsythia* (*T.f.*), *Treponema denticola* (*T.d.*) and *Enterococcus faecalis* (*E.f.*).

**Results:**

Among all samples, F.n. was most common (98%), followed by *T.f.* (73%), T.d. (66%), *P.g.* (45%), *A.a.* (12%) and *E.f.* (3%). Controls showed no *P.g.*, *A.a.* and *E.f.*. In stage I *T.f.* (*p* < 0.001) and *T.d.* (*p* = 0.022) were significantly more frequent. In stages II and III, *P.g.*, T.f. and *T.d.* were significant (*p* < 0,001). In stage IV, *A.a.* (*p* = 0.003), *P.g*., *T.f.* and *T.d*. (*p* < 0.001) occurred with significant higher frequency. Grade C was more common among stage III and IV (20%) as compared to stage I and II (2.5%). Patients diagnosed with stage IV and grade C, showed significant association to *A.a.* (*p* = 0.001) and *P.g.* (*p* = 0.021).

**Conclusions:**

The present investigation proved significant correlation between periodontopathogenic key species, disease severity and progression risk. *A.a.* and *P.g* were most frequently in stage IV and grade C.

**Clinical relevance:**

Microbial analysis provide additional information in regard to the classification of periodontitis.

## Introduction

Periodontitis has long been considered a non-specific infectious disease [1], but studies have shown that the composition of the subgingival microbial community and especially the occurrence of certain periodontopathogenic bacteria play a more important role [2–5]. It was realized that synergistic polymicrobial interactions have a modulatory effect on the host immune system and tip the change from homeostasis to dysbiosis [6–8]. Finally, a combination of microbial virulence and impaired immune surveillance leads to a self-destructive inflammatory response [6, 9, 10]. Additional risk factors, such as smoking, non-controlled diabetes, and the individuals’ own predisposition further contribute to the manifestation and progression of the disease [11–16].

In this regard, various studies have shown that the predominant microbiome of periodontitis patients significantly differs from that of healthy subjects or individuals diagnosed with gingivitis only [17–19].

In this context, research by Sokransky and co-workers, led to the identification of five bacterial complexes that repeatedly coexist within the subgingival biofilms of periodontitis patients. Complexes that include the most virulent pathogens comprise the Gram-negative anaerobic species *Porphormonas gingivalis* (*P.g.*), *Tannerella forsythia* (*T.f.*) and *Treponema denticola* (*T.d.*) [20]. The emerging of these highly pathogenic bacteria is characteristic for mature subgingival biofilms and relies on the presence of earlier species such as *Fusobacterium nucleatum* (*F.n.*) [21–25]. As shown, there is also significant association between the presence of these so-called red complex bacteria and clinical parameters such as periodontal pocket depth and attachment loss [26, 27]. In particular, it was shown that especially the emerging of *Porphyromonas gingivalis* is strongly correlated with disease progression [28, 29]. Furthermore, some virulent genotypes of the Gram-negative species *Aggregatibacter actinomycetemcomitans* (*A.a.*) are also associated with high risk of early and rapid periodontal tissue destruction [30–32]. Thus, present data suggests that the prevalence of *Enterococcus faecalis* (*E.f.*) in periodontitis patients is also higher compared to periodontally healthy individuals [33].

From a diagnostic and therapeutic point of view, classification systems are important measures. In the case of periodontitis, a new classification scheme was recently introduced which enables a uniform disease characterization in clinical practice, research and epidemiological surveillance [34].

The recent established system relies on a staging and grading approach and allows the assessment of the severity, complexity and progression risk in a detailed and standardized manner [35]. Disease severity and complexity is now defined by four different stages that are based on clinical and radiographic findings: initial (stage I), moderate (stage II), severe (stage III) and very severe (stage IV) [34]. The grade of periodontitis is estimated with direct or indirect evidence of disease progression and is expressed by three different values: grade A - low, grade B - moderate, grade C– high. Estimation of the periodontitis grades includes a history-based analysis of the periodontitis progression rate, bone resorption index, assessment of progression risk factors such as smoking or bad blood sugar control in diabetes patients, and an assessment of general health issues that have a negative impact on the therapy and treatment outcome [34, 35].

Unfortunately, characterization of the microbiome is not yet a subject of the current classification system. Correlations are already observed, but information in association to stages and grades is still insufficient [36–40]. Therefore, the present investigation aimed on identifying selected key species (*Fusobacterium nucleatum*, *Aggregatibacter actinomycetemcomitans*, *Porphyromonas gingivalis*, *Tanerella forsythia*, *Treponema denticola*, *Enterococcus faecalis)* in association to the current classification of periodontitis, in particular with focus on the periodontitis prognosis assessment.

## Materials and methods

### Patients

The present clinical and microbiological examination compromised a total of 100 patients, recruited by the Department of Conservative Dentistry and Periodontology, University Hospitals, Jena, Germany. The study was approved by a local ethical committee (Ethic Committee, Medical Faculty, Friedrich-Schiller University, Bachstraße 18, 07743 Jena, Germany; ID: 2019 − 1566) and written informed consent was given. The patients were assigned to 4 different test groups (periodontitis stage I-IV) and one healthy control group. A sample size of 20 participants in each group was previously calculated by a power analysis (85%), applying a significance level of 5%.

The study included only adults diagnosed with either periodontitis (test groups I-IV)), or healthy subjects with a max. probing depth of 3 mm and BoP < 10% (control group). Periodontitis was diagnosed based on clinical and radiographic signs in accordance to the classification of periodontitis. Patients with generalized gingivitis, smokers, addicted to drugs, pregnancy, edentulous patients, infectious diseases, severe general illnesses and patients who have received antibiotic treatment during the last 4 months were excluded from the study. Patients were examined from 05/2022 to 07/2023, before undergoing periodontitis 2-step therapy [41].

### General medical history

Information on general diseases were collected that have a major influence on periodontitis. This also included an evaluation of the patients’ smoking behavior as well as regular medication intake, alcohol abuse and stress level.

### Dental status

The dental status of each patient was recorded in a preventive manner. This included a careful examination of all teeth and inspection of the oral mucosa. Further, the color, shape and surface texture of the gingiva was observed. Dental fillings were checked, and carious lesions, destroyed or missing teeth were recorded. Conservative, prosthetic and endodontic therapies were assessed with regard to their sufficiency.

### Periodontitis anamnesis

A detailed survey in accordance to the patient’s subjective complaints such as bad breath, gum bleeding, tooth hypermobility, tooth loss due to periodontitis, tooth migration or pain has been carried out. The patients were also asked for previous periodontitis therapies and follow-up treatments.

### Periodontal-screening-index (PSI)

The maximum pocket depth of each sextant was diagnosed using a WHO calibrated periodontal probe. Probing depths of up to 3.5 mm were considered healthy (code 0). At codes 1 and 2, although there are no pathological pockets, there are clinical signs of gingivitis. Periodontitis was assessed for probing depths exceeding 3.5 mm (code 3) and 5.5 mm (code 4).

### Bleeding on probing (BoP)

The number of bleeding-positive assessment points was recorded for the entire dentition using a blunt periodontal probe. According to the current classification scheme, if there is a threshold < 10%, the criterion of periodontal health is met. Subjects with a BoP < 10% were assigned to the control group.

### Clinical attachment level (CAL)

CAL was determined by measuring periodontal pocket depths at six assessment points per tooth using a millimeter graduated periodontal probe. Also, furcation involvement (grades I-III), tooth hypermobility (grades I-III) and gingival recession levels were recorded.

### Radiographic examination

Panoramic radiographs were taken and radiographic bone loss (RBL) as a percentage of the total root length and total bone highs in relation to the patients’ age (bone loss index) was determined.

### Group allocation

According to the current classification of periodontitis, patients were assigned to 4 different test groups:

**Table:** 

test group 1 (Stage I, mild disease)	*n* = 20
test group 2 (Stage II, moderate disease)	*n* = 20
test group 3 (Stage III, severe disease)	*n* = 20
test group 4 (Stage IV, advanced disease)	*n* = 20
healthy subjects (control group)	*n* = 20

### Microbial sampling

Samples were collected from the deepest periodontal pockets of each sextant using sterile paper points (white, Roeko Coltene). The paper points were inserted in the periodontal pockets for 15 s and afterwards transferred to an Eppendorf reaction vessel. From each patient 6 paper points were obtained and pooled. Control samples were collected identically. All samples were stored at 4 °C until use.

### PCR analysis

Samples were analyzed by PCR using the Thermocycler MasterCycler Realplex (Eppendorf Vertrieb Deutschland GmbH/ WesselingBerzdorf / Germany). All analyses were performed at the Oral-biological laboratory, Department of Conservative Dentistry and Periodontology, Jena University Hospital, Germany. First, each paper point was transferred to a single sterile Eppendorf tube containing 100 µl of PCR grade water, vortexed for 3 min and subsequently denaturated at 99 °C for 10 min.

Cooled samples were centrifuged at 14,000 rpm for 2 min and aliquots of 150 µl were transferred to empty reaction vessels. Four microliter of each suspension was then mixed with 21 µl reaction media (5 µl PCR puffer, 1.5 µl MgCl2 (25 mM), 13.8 µl PCR grade water, 0.5 µl dNTP-Mix (10 mM ), 0.05 µl Primer 3´forward ( 100 µM ), 0.05 µl Primer 5` reverse ( 100 µM ), 0.12 µl Taq Polymerase (5U /µl)).

Samples were analyzed for *Fusobacterium nucleatum (F.n.)*, *Aggregatibacter actinomycetemcomitans (A.a.)*, *Porphyromonas gingivalis (P.g.)*, *Tanerella forsythia (T.f.)*, *Treponema denticola (T.d.)* and *Enterococcus faecalis (E.f.)*.

### Statistical analysis

Data was analyzed using the statistical software program SPSS version 27.0. Determination of the relative frequency, calculation of means and medians as well as the Mann-Whitney U test were part of the descriptive analysis. Further, Kruskal-Wallis-test was used for comparing means. Inductive statistics included Fischer’s exact test which was used when conditions for the chi-square test were not met. Furthermore, odds were calculated by using a binary logistic regression model. A 5% significance level and a 95% confidence interval were applied. Significance was obtained for *p* < 0.05. For pairwise comparison, significance was adjusted in accordance to Bonferroni.

## Results

The study included 45 male and 55 female participants with mean ages ranging between 40 (test group 1), 59 (test group 2), 55.5 (test group 3), 55 (test group 4) and 31.5 years (control group). There was no significant difference between male and female participants in regard to the periodontitis stage allocation (*p* = 0.52, Pearson-Chi-Quadrat).

It was found that periodontal pocket depth significantly increased by stage (*p* < 0.001; Kruskal-Wallis-Test). When compared to the healthy control patients, significant differences in the mean pocket depth were assessed for stage II, III and IV. Significance was also obtained when stage I was compared to the stages II, III and IV and for stages III and IV in regard to stage II (Table 1).

**Table 1:** An increase in disease severity is associated with an increase in pocket depths. There is significant differences for stage II-IV when compared to the healthy controls

	test group. 1 (stage I)	test group 2 (stage II)	test group 3 (stage III)	test group 4 (stage IV)	control group (0)
Mean pocket depth (mm)	4	5	7	10	2
interquartile range	0	0	2	7	1
		*p* = 0.001 (Kruskal-Wallis-Test)			

Mean periodontal pocket depth (mm)

### Microbial assessment

Among all samples (*n* = 100), *F.n.* was detected most frequently (98%), followed by *T.f.* (73%), *T.d.* (66%), *P.g.* (45%), *A.a.* (12%) and *E.f.* (3%). In all subjects (periodontitis patients, healthy control patients), the bacteria *F.n.*, *T.f.* and *T.d.* were present. Patients diagnosed with periodontitis (test groups 1–4) additionally showed *A.a.*, *P.g.* and *E.f.* which were not detected in the healthy control group. Table 2 shows the relative frequency of positive PCR results for each group.

**Table 2:** Results are dominated by *F.n.* The species *E.* f. was detected with the lowest Frequency. In the control group *A.a.*,.P.g and *E.f.* were not present

	F.n.	A.a.	P.g.	T.d.	T.f.	E.f.
Test group 1	100%	0%	10%	60%	70%	0%
Test group 2	100%	10%	55%	80%	85%	5%
Test group 3	100%	10%	70%	85%	100%	5%
Test group 4	100%	40%	90%	85%	100%	5%
Control group	90%	0%	0%	20%	10%	0%

Relative frequency of positive PCR results

It was shown that *T.f.* (*p* < 0.001) and *T.d.* (*p* = 0.022) were significantly more frequent in test group 1 (stage I periodontitis) when compared to the control group. In test group 2 and 3 (stage II and stage III), the bacteria *P.g.*, *T.f.* and *T.d.* (*p* < 0,001) were detected at significant levels. In test group 4 (stage IV), *A.a.* (*p* = 0.003), *P.g.*, *T.f.* and *T.d.* (*p* < 0.001) were detected most commonly.

Significant differences were also found between the single periodontitis stages. Especially for test group 4 (stage IV) significant higher rates in *A.a.*, *P.g.*, and *T.f.* were detected compared to test group 1 (stage I). In test group 4 (stage VI), *P.g.* was also found more frequently compared to test group 2 (stage II). In test group 3 (stage III), *P.g.* and T.f. were detected to significant higher extents compared to test group 2 (stage II). *P.g.* was detected more frequently in test group 2 (stage II) compared to test group 1 (stage I). Pairwise comparison is summarized in Table 3.

**Table 3:** Inter-group comparison of relative frequency of positive PCR results. Significance is shown in bold

	*Chi-squared test (Fisher’s exact test); p < 0.05*					
group comparison	F.n.	A.a.	P.g.	T.f.	T.d.	E.f.
test group 1 / control	0.487	-	0.487	**< 0.001**	**0.022**	-
test group 2 / control	0.487	0.487	**< 0.001**	**< 0.001**	**< 0.001**	1.000
test group 3 / control	0.487	0.487	**< 0.001**	**< 0.001**	**< 0.001**	1.000
test group 4 / control	0.487	**0.003**	**< 0.001**	**< 0.001**	**< 0.001**	1.000
test group 2 / test group 1	-	0.487	0.006	0.451	0.301	1.000
test group 3 / test group 1	-	0.487	**< 0.001**	**0.02**	0.155	1.000
test group 4 / test group 1	-	**0.003**	**< 0.001**	**0.02**	0.155	1.000
test group 3 / test group 2	-	1	0.514	0.231	1	1.000
test group 4 / test group 2	0.065	**0.031**	0.231	1	1.000	
test group 4 / test group 3	0.065	0.235	1	1.000		

Inter-group comparison of bacterial frequencies

In Table 4 relative frequencies of all positive PCR results are listed. Among the test groups (periodontitis stages I-IV) all investigated species were present, while there was no proof of *A.a.*, *P.g.* and *E.f.* in the control group. *F.n.* was the most frequent species (90%) found in the control group, followed by *T.d.* (20%) and *T.f.* (10%). Among the periodontitis patients *F.n.* (100%), followed by *T.f.* (88.8%) and *T.d*. (77.5%) showed the highest frequencies.

**Table 4:** Relative frequency of positive bacterial results in test groups 1–4 (with stages I-IV) and the control group. Significance was indicated by bold numbers

	test groups 1–4 (St. I-IV)	control group	*Fisher’s exact test*, *p < 0.05*
**F.n.**	100%	90%	**0.038**
**A.a.**	15%	0%	0.117
**P.g.**	56%	0%	**0.001**
**T.f.**	88.8%	10%	**0.001**
**T.d.**	77.5%	20%	**0.001**
**E.f.**	4%	0%	1

Combined relative frequencies of all test groups in comparison to the control group

### Grading

It was shown that there is an increase in the frequency of grade B and especially of grade C in stage III and IV patients (Table 5). In comparison, grade A was much stronger associated with test group 1 and 2. The distribution of grade A-C was significant (*p* < 0.001). When stage I and II as well as III and IV were grouped together, grade C was more commonly detected among periodontitis stages III and IV (20%) as compared to stages I and II (2.5%). The results were significant (*p* = 0.029).

**Table 5:** Grade distribution among the test groups. Grade C was significantly associated with test groups 3 and 4 (bold)

	grade A	grade B	grade C	Freeman-Halton test, for grades A-C; *p* < 0.05	Fisher’s exact test, for grade A, B vs. C; *p* < 0.05
**test group 1** (stage I)	80%	15%	5%	*p* < 0.001	2.5%
**test group 2** (stage II)	80%	20%	0%		
**test group 3** (stage III)	45%	40%	15%		20% *p* = 0,**029**
**test group 4** (stage IV)	15%	60%	25&		

Periodontitis prognoses assessment, grade distribution

In test group 4 (stage IV) and grade C, the species *A.a.* and *P.g.* were detected more frequently (Table 6). The significance for *A.a.* (*p* = 0.001) was even stronger compared to *P.g.* (*p* = 0.021).

**Table 6:** Relative frequency of positive (1) and negative (0) bacterial results in test group 4 (stage IV) and grade C compared to the remaining test groups (stages I-III) and grades < c. *A.a.* and *P.g.* show significant association to stage IV, grade C (bold)

		stage ≤ IV or grade < C	stage IV and grade C	Fisher’s exact test; *p* < 0.05
F.n.	1	91,8%	8,2%	1.000
	0	100%	0	
A.a.	1	50%	50%	**0.001**
	0	97.7%	2.3%	
P.g.	1	84.4%	15.6%	**0.021**
	0	98.2%	1.8%	
T.d.	1	89.4%	10.6%	0.259
	0	97.1%	1.9%	
T.f.	1	89.0%	11%	0.104
	0	100%	0	
E.f.	1	100%	0	1.000
	0	91.8	8.2	

Bacterial frequencies in stage IV, grade C patients

## Discussion

The data of the present in-vivo-study shows that there is qualitative correlation between the occurrence of peridontopathogenic key species and the patients’ classification stage. Based on the evaluated data, it is evident that the two species *A. actinomycetemcomitans* and *P. gingivalis* were significantly associated with stage IV (advanced periodontitis). Among stage IV patients, *A.a.* and *P.g.* were also significantly more likely in grade C (high progression rate) as compared to the grades A or B (slow and moderate progression risk).

In the present investigation, *F. nucleatum* was significantly more common among periodontitis patients (stage I-IV) compared to healthy subjects (control group). Likewise, species of the red complex (P.g., *T. f.* and *T. d.*) were also detected with significant higher frequency in moderate to severe cases as compared to healthy individuals. These results partly correlate to recently published data that reports on high levels of red complex *T. denticola* and a moderate increase in orange complex microorganisms in patients with severe periodontitis [40, 42].

In the present study, there was also a significant distribution of the progression grades (A-C) among the individual periodontitis classification stages. Grade C was detected more often in the stages III and IV as compared to stage I and II. Additionally, the measured probing depths significantly increased by disease severity. It has been shown that an increase in periodontal pathogens and the prevalence of subgingival dysbiosis correlates to an increase in probing depth and, thus, severity of periodontitis [43, 44].

In this context, the present study revealed that *A.a.* was also detected to a significant greater amount in stage III and IV (mean probing depth of 7 and 10 mm) patients. This is in line with results of a former study that investigated the occurrence of *A.a*. in patients with aggressive periodontitis [45]. In other studies, *A.a.* was also significantly more common in patients with deeper pockets as compared to those with less severe probing depths [46–49].

In the present investigation, red complex species (*P.g*., *T.f*. and *T.d.*) were identified more frequently among patients with increased pocket depths, too. For pockets < 4 mm, these species were less likely to be found compared to pockets between 4 and 6 mm. Red complex species were most commonly investigated in pockets > 6 mm. This is in line with findings of other authors that also identified these species in subgingival plaque samples of patients with increased probing depths [28, 50].

The results of the present study revealed that in patients with stage IV (severe periodontitis), the bacterial species *A.a.* and *P.g.* were detected to a significant higher frequency as compared to all other stages. In healthy subjects (control group), *A.a.* and *P.g.* were not detected at all. Similar results were also reported by another recently published investigation [47]. It was found that patients with severe periodontitis (stage III/IV, grade C), before and 6 months after non-surgical therapy with adjuvant antibiotic treatment showed high levels of *P.g.* The authors concluded that *P.g.* and also *A.a.* are rated key species for severe periodontitis [49]. Silva et al. also discussed an increased frequency of *A.a.* and *P.g.* at sites with progressive attachment loss [51]. Thus, even in small quantities, *P.g.* was significantly associated with severe periodontitis, while *A.a.* was assessed only at a certain threshold [52].

The results of the present investiagtion shows that there is a significant higher occurrence of *A.a.* in stage IV, compared to the stages I-III, but still in lower quantity compared to the species *F.n.*, *P.g.*, *T.f.* and *T.d.*.

In this regard, *A.a.* was diagnosed only in 12 out of 100 subgingival plaque samples. 66.7% of the positive findings were associated with stage IV and only 16.7% with stage III and II. In addition, it has been shown that *A.a.* is not only significantly more likely to be associated with stage IV but also with a higher progression risk (grade C). This was also confirmed for *P.g.*, but to a less significant extent.

For *A.a.*, there are some interesting studies that show that presumably specific clones of this species are strongly associated with aggressive periodontitis. This includes the highly leucotoxin-producing JP2 clone, which is associated with rapid disease progression [53–55].

Despite a high relative abundance within all groups, *F.n.* was identified significantly more common in periodontitis patients (test groups 1–4) as compared to periodontal healthy individuals. Other studies also discussed that *F.n.* can be found, both, in periodontitis patients and also in healthy subjects [56, 57].

It is known that *F.n.* can adhere directly to epithelial cells by various adhesins. Because its ability to co-aggregate also with other pathogenic species, *F.n.* becomes a “connector” to bacteria of the red complex and thus contributes to the development of pathogenic periodontal biofilms [58, 59].

Due to its mediating role and capability in interacting with other pathogenic species, *F.n.* plays a key role in the “shift” within the subgingival microbiome to a predominance of other strictly anaerobic bacteria and, thus, contributes to the establishment of microbial dysbiosis [23]. Research of our own study group has shown that photodynamic treatment efficiently suppresses *F.n.*, causing a significant reduction in inflammatory signs [60–62].

In recent time, *F.n.* has increasingly become a subject of research, also due to a connection with extraoral diseases such as cardiovascular diseases, intestinal inflammatory diseases, gastrointestinal cancers, breast and colorectal cancer, Alzheimer’s disease, rheumatoid arthritis, and respiratory diseases [63–66]. The species is also common in the oral microflora of periodontal healthy individuals [23, 67]. This is also shown by the high proportion of *F.n.* positive results in the recent control group. Besides *F.n.*, the gram-positive species *E. faecalis* was also observed in the present study. Out of 100 participants, *E.f.* was found only in three periodontitis patients assigned to the stages II, III and IV. It is known that *E.f.* is responsible for the transmission of virulence factors and might be associated also with therapy-refractory cases [68–70].

In the recent periodontitis classification system, microbial diagnostics has not yet been taken into account. Until now, diagnosis and therapeutic measures are only based on clinical and radiographic findings. However, subgingival bacterial profiles significantly differ between disease and periodontal health and, thus, can also underline an initial risk assessment [18]. In addition, microbial evaluations can serve as indicator for an adjuvant antibiotic concomitant treatment. Microbial analysis further help to monitor the success of a treatment and support the identification of refractory cases.

## Conclusions

The present investigation proved that the periodontopathogenic key species *Aggregatibacter actinomycetemcomitans* and *Porphyromonas gingivalis* are strongly associated with stage IV and grade C. It can be concluded that the combined occurrence of both species is an indicator for unfavorable courses. Due to the present results, microbial assessments might become significantly more important in future. In the author’s opinion, microbial evaluations are useful and should be considered also by the current periodontitis classification system.

## Data Availability

No datasets were generated or analysed during the current study.
